# Unveiling the relationship between dietary patterns and sleep quality: cross-sectional evidence from university students in Jinan city

**DOI:** 10.3389/fnut.2026.1797372

**Published:** 2026-04-02

**Authors:** Zimeng Han, Zefang Li, Wenqi Liu, Xueqiang Wu, Yuan Tian

**Affiliations:** 1 School of Nursing, Shandong University of Traditional Chinese Medicine, Jinan, China; 2 College of Traditional Chinese Medicine, Shandong University of Traditional Chinese Medicine, Jinan, China; 3 First Clinical Medical College, Shandong University of Traditional Chinese Medicine, Jinan, China; 4 School of Foreign Languages, Shandong University of Traditional Chinese Medicine, Jinan, China

**Keywords:** Chinese university students, Cross-sectional study, dietary pattern, Principal component analysis, sleep quality

## Abstract

**Objective:**

Poor sleep quality is common among university students, yet its association with overall dietary patterns remains unclear. This research investigated how dietary patterns relate to poor sleep quality in a population of Chinese university students, using a cross-sectional design.

**Methods:**

From September to December 2024, we conducted a cross-sectional study at two universities in Jinan, Shandong Province, China. A total of 1,497 students were recruited using cluster sampling. Dietary intake was assessed using a validated Food Frequency Questionnaire (FFQ) covering 12 major food groups. Dietary patterns were derived using principal component analysis (PCA). Sleep quality was assessed using the Pittsburgh Sleep Quality Index (PSQI). Multivariable logistic regression was used to examine associations between dietary pattern scores and poor sleep quality.

**Results:**

Three dietary patterns were identified. In fully adjusted models, each 1-SD increase in the PCA3 score was associated with lower odds of poor sleep quality OR = 0.81 (95% CI: 0.70 to 0.93). A significant dose-response trend was observed across PCA3 quintiles (*P* for trend = 0.001), and associations were generally consistent across demographic and mental health subgroups. No significant associations were observed for the PCA1 or the PCA2.

**Conclusion:**

Higher adherence to the PCA3-characterized by higher intakes of fresh fruits, fresh vegetables, and dairy products-was associated with lower odds of poor sleep quality among Chinese university students.

## 1 Introduction

Sleep quality is a core dimension of sleep health and is associated with cognitive performance, emotional regulation, and overall wellbeing (1). University students are particularly vulnerable to poor sleep quality due to academic pressure, irregular schedules, and lifestyle transitions in early adulthood (2–4). In China, survey evidence indicates that poor sleep quality are common among Chinese university students (5). Poor sleep quality may manifest as difficulty initiating sleep, fragmented sleep, early morning awakening, and daytime sleepiness (6, 7), and it is influenced by multiple factors, including demographic characteristics, lifestyle behaviors, psychological status, and health conditions (8). Persistent poor sleep quality is associated with increased fatigue, difficulty concentrating, and a decline in academic performance among university students (9). Moreover, evidence indicates a significant association between poor sleep quality and the worsening of cognitive function, endocrine dysregulation, and mental health issues in this population (3, 10–13). Identifying modifiable correlates of sleep quality in university settings is therefore important for health promotion and disease prevention.

Diet is a modifiable lifestyle factor that may contribute to sleep quality. While prior studies have often focused on single nutrients or individual foods (14–16), these approaches may not reflect real-world eating behaviors, in which foods are consumed in combination and within an overall dietary context. Dietary pattern analysis addresses this limitation by capturing habitual dietary structure and potential synergistic effects across foods, thereby offering evidence that is often more etiologically informative and relevant to population-level interventions. Existing studies suggest that sleep health is intertwined with metabolic regulation (17) and that poorer sleep tends to cluster with less healthy eating behaviors. Accordingly, Western-style dietary patterns characterized by higher intakes of processed meats, refined grains, and sugar-sweetened beverages have been associated with poorer sleep quality in some studies (18, 19), whereas plant-forward patterns have been linked to better sleep parameters (20–22). However, findings are not fully consistent, and most evidence comes from Western populations, which may limit generalizability to Chinese university students with distinct dietary structures and sociocultural contexts.

China exhibits substantial regional heterogeneity in dietary practices, reflected in differences in staple foods, culinary methods, and the relative contributions of animal-source foods across areas. Concurrently, dietary behaviors among Chinese young adults are undergoing rapid change as part of the ongoing nutrition transition, with greater availability and consumption of energy-dense foods high in salt, sugar, and fat (23). In parallel, a plant-forward eating pattern is emerging among some young people, characterized by higher intakes of fresh fruits and vegetables with continued consumption of dairy products, alongside lower intakes of meat and processed foods (24). These shifts are relevant to sleep quality because dietary structure may influence sleep–wake regulation through interconnected pathways, including metabolic homeostasis, inflammatory signaling, and neurotransmitter synthesis (21). However, evidence linking empirically derived dietary patterns to sleep quality among Chinese university students remains limited, particularly for locally relevant patterns that jointly capture plant foods and dairy.

To address this gap, we conducted a cross-sectional study among Chinese university students and used principal component analysis to derive major dietary patterns from food frequency data. Subsequently, we assessed sleep quality using the Pittsburgh Sleep Quality Index (PSQI). Poor sleep quality was defined as a global PSQI score >7 (25). We hypothesized that a dietary pattern characterized by higher intakes of fruits, vegetables, and dairy products would be associated with lower odds of poor sleep quality, and that this association would persist after accounting for psychological distress and disordered eating risk. By clarifying diet–sleep relationships at the dietary-pattern level in this population, this study aims to provide evidence relevant to dietary strategies for sleep health promotion in university settings.

## 2 Materials and methods

### 2.1 Study population

A cross-sectional survey was conducted among Chinese university students in Jinan City, Shandong Province, China, from September to December 2024 to examine the association between dietary patterns and sleep quality. Participants were recruited using cluster sampling from two universities in Jinan, with classes serving as the primary sampling units. Trained investigators visited classrooms to invite students to participate and administered paper-based questionnaires to collect information on sociodemographic characteristics, sleep quality, dietary intake, mental health, and other potential covariates. A total of 1,497 students aged 17–26 years completed the survey and were included in the analysis.

### 2.2 Food frequency assessment

Dietary intake was assessed using a Food Frequency Questionnaire (FFQ) adapted from the China Kadoorie Biobank questionnaire (26). The FFQ has demonstrated acceptable reliability and validity in Chinese populations (27). It included 12 commonly consumed food groups: rice, wheat products, other staple foods (e.g., corn and millet), meat, poultry, fish/seafood, eggs, dairy products, fresh vegetables, preserved vegetables, fresh fruits, and other dairy products. Participants reported their average consumption frequency for each food group over the past year. Frequency categories were converted to days per week as follows: never or rarely (0), monthly (0.5), 1–3 days/week (2), 4–6 days/week (5), and daily (7) (28).

### 2.3 Sleep quality evaluation

We evaluated sleep quality using the PSQI (25). Seven component scores are generated from the 19 self-rated items comprising the PSQI, with each scored from 0 to 3. The global PSQI score (range: 0–21; higher scores reflect worse sleep quality) was calculated by summing the seven component scores. Following established criteria (29), we classified participants with a PSQI total score >7 as having poor sleep quality, thereby creating poor- and good-sleep-quality groups. In secondary analyses, we also examined the association between dietary pattern scores and individual PSQI component scores.

### 2.4 Covariates

We considered a range of potential confounders selected *a priori* based on prior literature. Participants reported demographic and socioeconomic characteristics through questionnaire completion, primarily including age, sex, ethnicity, education, household income, and household size (20, 30, 31). Disordered eating risk was assessed using the Eating Attitudes Test (EAT-26); a total score ≥20 was used to indicate elevated risk of an eating disorder (32–34). Psychological distress was assessed using the 21-item Depression Anxiety Stress Scales (DASS-21) (35–37). Body mass index (BMI) was calculated using the formula: weight (kg)/height (m)^2^ (38). Among the lifestyle factors assessed were smoking status and alcohol consumption (39). Participants did not use sedative-hypnotic drugs. Covariates are presented in Table 1.

**Table 1:** Baseline characteristics of participants by sleep quality status.

Variables	Good sleep quality group, n (%)	Poor sleep quality group, n (%)	*P*-value
Number of participants	1,093	404	
Sex			
Women	811 (74.2)	325 (80.4)	0.012
Men	282 (25.8)	79 (19.6)	
Age, mean (SD)	19.0 (1.4)	19.3 (1.8)	0.002
BMI			
< 18.5	236 (21.6)	69 (17.1)	0.078
18.5 to 25	691 (63.2)	260 (64.4)	
≥25	166 (15.2)	75 (18.6)	
Education			
Master's degree	32 (2.9)	22 (5.4)	0.002
Bachelor's degree	650 (59.5)	263 (65.1)	
Associate's degree	411 (37.6)	119 (29.5)	
Smoke			
Never	1,062 (97.2)	397 (98.3)	0.453
Past	15 (1.4)	4 (1.0)	
Present	16 (1.5)	3 (0.7)	
Drink			
Never	956 (87.5)	349 (86.4)	0.511
Past	83 (7.6)	29 (7.2)	
Present	54 (4.9)	26 (6.4)	
Ethnicity			
Han	1,085 (99.3)	393 (97.3)	0.002
Other	8 (0.7)	11 (2.7)	
Medical history			
No	1,077 (98.5)	395 (97.8)	0.306
Yes	16 (1.5)	9 (2.2)	
Medication/supplement use			
No	1,080 (98.8)	394 (97.5)	0.073
Yes	13 (1.2)	10 (2.5)	
Family structure			
Nuclear family	934 (85.5)	328 (81.2)	0.233
Extended family	101 (9.2)	47 (11.6)	
Single-parent family	12 (1.1)	7 (1.7)	
Blended family	46 (4.2)	22 (5.4)	
Household size, mean (SD)	4.1 (1.0)	4.1 (0.9)	0.370
Household income, mean (SD)	10.4 (18.4)	11.9 (29.1)	0.359
EAT-26			
< 20	1,018 (93.1)	318 (78.7)	< 0.001
≥20	75 (6.9)	86 (21.3)	
DASS-Stress			
No	929 (85.0)	167 (41.3)	< 0.001
Yes	164 (15.0)	237 (58.7)	
DASS-Anxiety			
No	781 (71.5)	116 (28.7)	< 0.001
Yes	312 (28.5)	288 (71.3)	
DASS-Depression			
No	953 (87.2)	200 (49.5)	< 0.001
Yes	140 (12.8)	204 (50.5)	

### 2.5 Statistical analysis

Continuous variables are expressed as means with standard deviations (SD), while categorical variables are reported as frequencies and percentages. To assess differences between the two groups defined by sleep quality (poor vs. good), continuous variables were analyzed with one-way ANOVA, and categorical variables were analyzed using the chi-square test. In cases where contingency tables had expected cell counts below five, Fisher's exact test was applied instead.

In order to identify potential dietary patterns within the study population holistically, we employed principal component analysis (PCA) to reduce the dimensionality of consumption frequency data across 12 food categories. For dietary pattern derivation, the 12 food-group frequency variables were standardized (z-scores) prior to principal component analysis to improve comparability across food groups. Inflection points were identified based on eigenvalues and scree plots (Supplementary Figure S1) to jointly determine the quantity of principal components to retain. Subsequently, to improve interpretability, a variance-maximizing rotation was subsequently applied to the extracted components (40–42). The resulting patterns were named based on food groups with high factor loadings, with food groups with an absolute load value ≥0.60 are designated as primary contributors, and each participant's adherence was quantified using corresponding factor scores. We assessed the suitability of the data for factor analysis using the Kaiser–Meyer–Olkin (KMO) measure of sampling adequacy and Bartlett's test of sphericity. The KMO measure and Bartlett's test (Supplementary Table S1), detailed factor loading matrix (Supplementary Table S2) is presented in Supplementary material. Binary logistic regression was used to estimate odds ratios (ORs) and 95% confidence intervals (CIs) for associations between dietary pattern scores and poor sleep quality. Sequential multivariable models were fitted: model 1 (adjusted for sex, age, ethnicity, and education); model 2 (further adjusted for smoking status, household size, alcohol consumption, household income, body mass index, EAT-26 score, and DASS-21 measures). Linear regression was employed to examine associations with individual PSQI component scores. Subgroup analyses assessed potential effect modification by including multiplicative interaction terms, and sensitivity analyses treated the global PSQI score as a continuous variable. All analyses were performed in R (version 4.5.2) using two-sided tests, with α = 0.05 set as the significance threshold. This study has been approved by the Ethics Committee of the Affiliated Hospital of Shandong University of Traditional Chinese Medicine (Approval No.: 2023-75-KY).

## 3 Results

### 3.1 Participant characteristics

Of the 1,497 participants, 27.0% (n=404) reported poor sleep quality. As shown in Table 1, compared to their counterparts with good sleep quality, participants with poor sleep quality demonstrated a higher proportion of women; a slightly higher mean age; a lower proportion of Han ethnicity; and a greater proportion with a master's degree (all *P* < 0.05). The poor sleep quality group also had higher prevalences of depressive, anxiety, and stress symptoms and a higher proportion of participants with disordered eating risk (EAT-26 ≥20; all *P* < 0.001). Comparisons of BMI category, smoking status, alcohol consumption, medical history, medication/supplement use, family type, household size, and income revealed no statistically significant intergroup differences (all *P* > 0.05). Supplementary Table S3 showed the association between FFQ and PSQI.

### 3.2 Dietary patterns derived by principal component analysis

**Figure 1:**
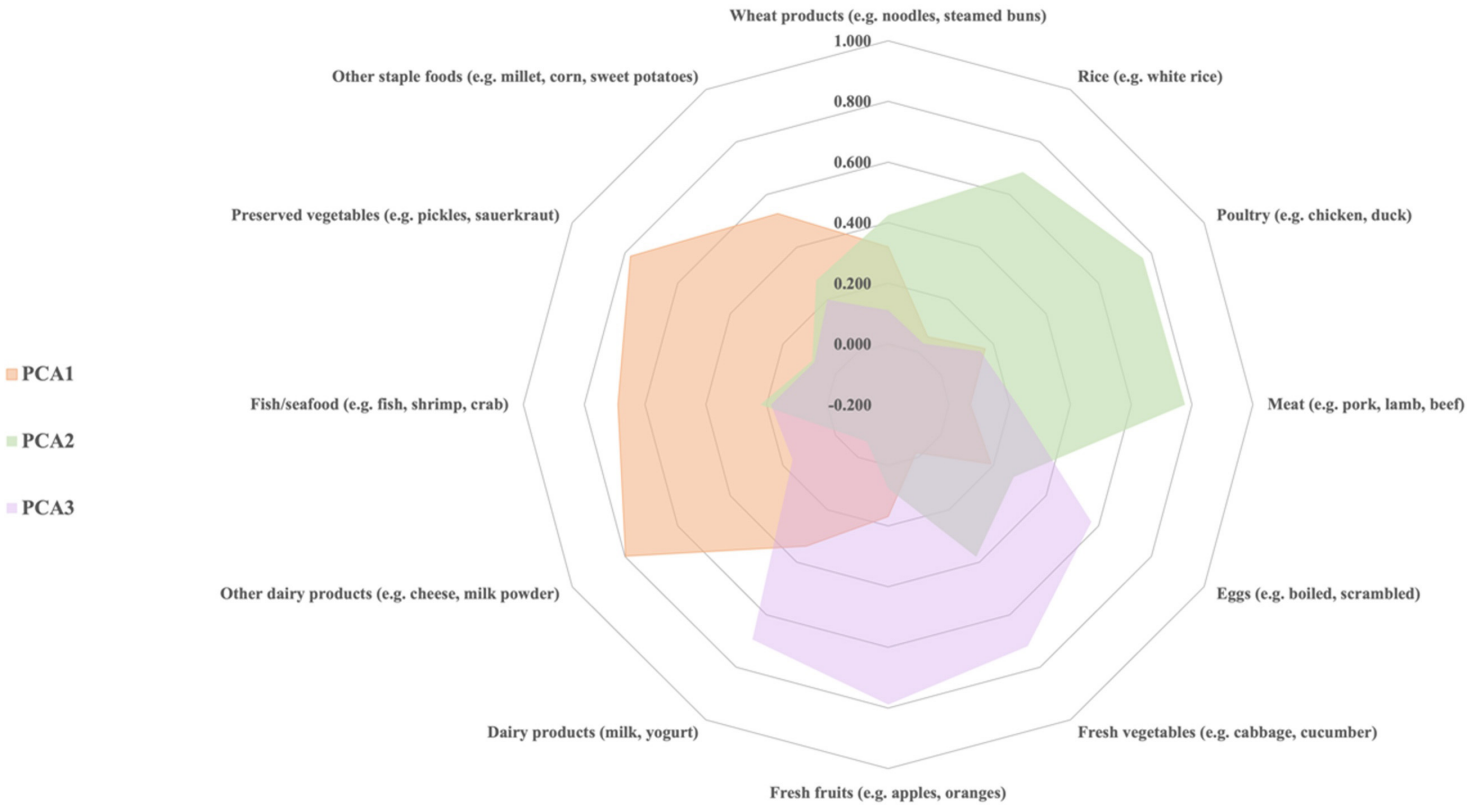
Rotated factor loading plot for dietary patterns derived from principal component analysis.

Three dietary patterns, namely the PCA1, the PCA2, and the PCA3, were identified through principal component analysis. The first pattern, termed the PCA1, was characterized by higher intakes of other dairy products, preserved vegetables, and fish/seafood. The second pattern, termed the PCA2, was characterized by higher intakes of meat, poultry, and rice. The third pattern, termed the PCA3, was characterized by higher intakes of fresh fruits, fresh vegetables, and dairy products (Figure 1).

### 3.3 Associations between dietary patterns and poor sleep quality

**Figure 2:**
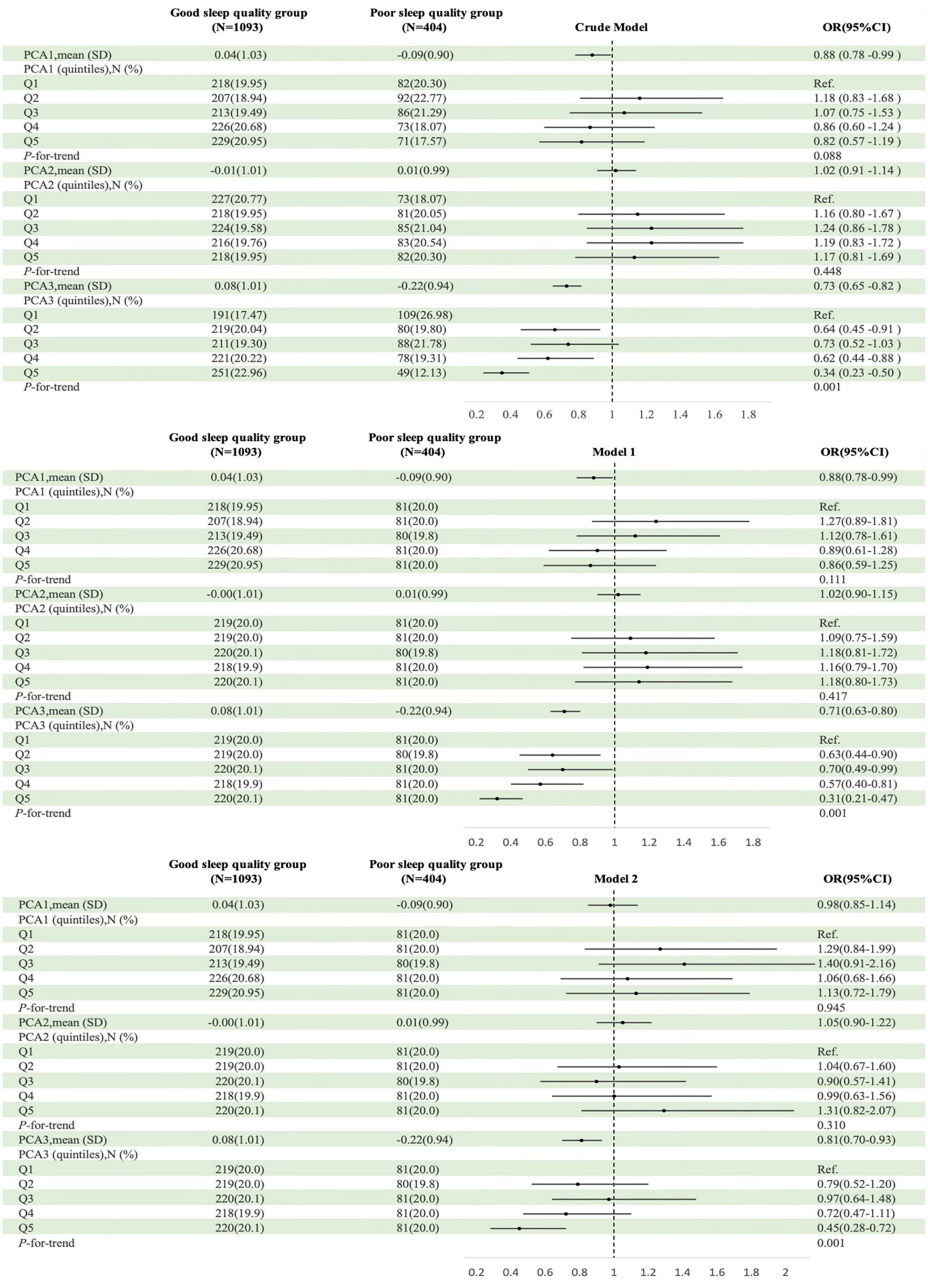
Associations between dietary pattern adherence and poor sleep quality. BMI, body mass index; CI, confidence interval; OR, odds ratio; SD, standard deviation; PCA1 (other dairy products, preserved vegetables, fish/seafood); PCA2 (meat, poultry, rice); PCA3 (fresh fruits, fresh vegetables, dairy products). Model 1 adjusted for sex, age, ethnicity, and education. Model 2 additionally adjusted for smoking status, household size, alcohol consumption, household income, BMI, and EAT-26 and DASS-21 scores. Quintiles of increasing dietary adherence (Q1–Q5).

No significant association between either the PCA1 or PCA2 and poor sleep quality was found in multivariable logistic regression analyses. In the fully adjusted model (Model 2), OR = 0.98 (95% CI: 0.85 to 1.14) for PCA1 and OR = 1.05 (95% CI: 0.90 to 1.22) for PCA2. Quintile analyses were consistent with these findings. In contrast, higher PCA3 score was linked to lower odds of poor sleep quality; in the fully adjusted model, each 1-SD increase in PCA3 score corresponded to an OR = 0.81 (95% CI: 0.70 to 0.93). Quintile analyses revealed a significant dose-response trend (*P* = 0.001), with participants in the highest quintile (Q5) exhibiting lower odds of poor sleep quality compared to those in the lowest quintile (Q1) OR = 0.45 (95% CI: 0.28 to 0.72; Figure 2).

### 3.4 Associations between PCA3 and PSQI component scores

To examine which aspects of sleep quality were most strongly associated with PCA3, we analyzed associations between PCA3 and the seven PSQI component scores (Table 2). In fully adjusted linear regression models, higher PCA3 score correlated with lower (better) scores for subjective sleep quality β = −0.05 (95% CI: −0.08 to −0.02), sleep latency β = −0.08 (95% CI: −0.11 to −0.04), sleep duration β = −0.04 (95% CI: −0.07 to −0.02), and daytime dysfunction β = −0.06 (95% CI: −0.10 to −0.02). Habitual sleep efficiency, sleep disturbances, and use of sleeping medication showed no significant associations.

**Table 2:** Associations between PCA3 adherence and PSQI component scores.

PSQI component	Overall, mean (SD)	Low PCA3 adherence, mean (SD)	Moderate PCA3 adherence, mean (SD)	High PCA3 adherence, mean (SD)	Fully adjusted β^a^ (95% CI)	*P*-value
Subjective sleep quality	0.61 (0.64)	0.66 (0.69)	0.67 (0.62)	0.51 (0.59)	−0.05 (−0.08, −0.02)	0.001
Sleep latency	0.57 (0.74)	0.68 (0.81)	0.58 (0.73)	0.44 (0.64)	−0.08 (−0.11, −0.04)	0.001
Sleep duration	0.36 (0.53)	0.42 (0.54)	0.36 (0.51)	0.31 (0.52)	−0.04 (−0.07, −0.02)	0.002
Habitual sleep efficiency	0.26 (0.56)	0.29 (0.58)	0.26 (0.54)	0.25 (0.55)	−0.02 (−0.05, 0.01)	0.181
Sleep disturbances	0.61 (0.58)	0.64 (0.62)	0.65 (0.56)	0.54 (0.56)	−0.01 (−0.04, 0.02)	0.386
Use of sleeping medication	0.03 (0.24)	0.04 (0.24)	0.03 (0.25)	0.03 (0.22)	−0.00 (−0.02, 0.01)	0.536
Daytime dysfunction	0.88 (0.96)	0.99 (1.03)	0.91 (0.93)	0.74 (0.90)	−0.06 (−0.10, −0.02)	0.004

### 3.5 Stratified analyses

With subgroup analyses (Table 3), the association between PCA3 and lower odds of poor sleep quality remained generally consistent across strata defined by sex, BMI category, EAT-26 score, and DASS-21 measures. The association appeared stronger among participants with BMI ≥25 kg/m^2^, OR = 0.61 (95% CI: 0.43 to 0.88) and among those with EAT-26 < 20, OR = 0.78, (95% CI: 0.67 to 0.92). Tests for interaction did not indicate statistically significant effect modification by stress (*P* = 0.865), anxiety (*P* = 0.906), depressive symptoms (*P* = 0.745), sex (*P* = 0.694), or BMI category (*P* = 0.377).

**Table 3:** Associations between PCA3 score (per 1-SD increase) and poor sleep quality across subgroups.

Subgroup	No. of participants, *n* (%)	PCA3 score (good sleep quality), mean (SD)	PCA3 score (poor sleep quality), mean (SD)	Fully adjusted OR^a^ (95% CI)	*P-*value
Sex					
Women	1,136 (75.89%)	0.10 (1.01)	−0.21 (0.93)	0.79 (0.68, 0.93)	0.005
Men	361 (24.11%)	0.04 (1.02)	−0.27 (0.99)	0.87 (0.63, 1.19)	0.388
*P*-for-interaction				0.694	
BMI					
<18.5	305 (20.37%)	0.07 (1.01)	−0.15 (0.97)	0.91 (0.63, 1.30)	0.602
18.5 to 25	951 (63.53%)	0.09 (1.00)	−0.18 (0.94)	0.83 (0.70, 0.99)	0.044
≥25	241 (16.10%)	0.08 (1.06)	−0.44 (0.90)	0.61 (0.43, 0.88)	0.009
*P*-for-interaction				0.377	
EAT-26					
<20	1,336 (89.25%)	0.08 (1.00)	−0.26 (0.94)	0.78 (0.67, 0.92)	0.002
≥20	161 (10.75%)	0.09 (1.11)	−0.10 (0.96)	0.99 (0.68, 1.44)	0.950
*P*-for-interaction				0.170	
DASS-stress					
No	1,096 (73.21%)	0.12 (1.01)	−0.06 (0.85)	0.80 (0.67, 0.97)	0.020
Yes	401 (26.79%)	−0.13 (0.97)	−0.34 (0.99)	0.80 (0.64, 1.00)	0.054
*P*-for-interaction				0.865	
DASS-anxiety					
No	897 (59.92%)	0.13 (1.00)	−0.02 (0.82)	0.77 (0.61, 0.96)	0.022
Yes	600 (40.08%)	−0.05 (1.02)	−0.30 (0.98)	0.81 (0.68, 0.98)	0.030
*P*-for-interaction				0.906	
DASS-depression					
No	1,153 (77.02%)	0.12 (1.00)	−0.08 (0.85)	0.79 (0.66, 0.94)	0.008
Yes	344 (22.98%)	−0.17 (1.04)	−0.36 (1.01)	0.82 (0.65, 1.04)	0.109
*P*-for-interaction				0.745	

### 3.6 Sensitivity analysis

In sensitivity analyses, higher PCA3 score remained associated with a lower PSQI total score in the fully adjusted model, β = −0.52 (95% CI: −0.75 to −0.29), with evidence of a dose-response trend across quintiles (*P* = 0.001). Supplementary Table S4 showed the detail of sensitivity Analysis.

## 4 Discussion

In this cross-sectional study of 1,497 Chinese university students, we identified three dietary patterns using principal component analysis and examined their associations with sleep quality. The key finding was an inverse association between adherence to the PCA3–characterized by higher intakes of fresh fruits, fresh vegetables, and dairy products—and poor sleep quality. This association was observed both when PCA3 was modeled continuously, OR = 0.81 (95% CI: 0.70 to 0.93), and across adherence quintiles OR = 0.45 (95% CI: 0.28 to 0.72), and it remained after adjustment for sociodemographic characteristics, lifestyle behaviors, adiposity, disordered eating risk, and psychological distress. Higher PCA3 scores were also associated with more favorable profiles in several PSQI domains—particularly subjective sleep quality, sleep latency, sleep duration, and daytime dysfunction—suggesting that PCA3 may relate to multiple dimensions of sleep health rather than a single symptom cluster. By contrast, neither the PCA1 nor the PCA2 showed statistically significant associations with poor sleep quality, whereas PCA3 demonstrated consistent associations across modeling approaches.

This study found that female students, older individuals, and graduate students generally exhibited poorer sleep quality, which is consistent with previous research. The underlying mechanisms may be related to hormonal fluctuations and academic stress (3, 7, 9). Our findings align with accumulating evidence that plant-forward dietary patterns are associated with more favorable sleep outcomes. A systematic review reported that dietary patterns characterized by higher intakes of fruits, vegetables, legumes, and foods containing melatonin- or tryptophan-related compounds are generally associated with better sleep quality (21), and observational studies have similarly suggested benefits of fruit- and vegetable-oriented patterns (43). Nevertheless, results across studies remain mixed; for example, a population-based Dutch cohort observed no significant association between dietary patterns and sleep quality (44). This heterogeneity may reflect differences in dietary structures across distinct populations. Studies indicate that Chinese university students are undergoing a transition in their dietary patterns, shifting from traditional plant-based diets to more complex and diversified eating habits (45). This change could influence the relationship between specific dietary patterns and sleep, thereby leading to results that differ from those observed in the Dutch study. In this context, our study adds data-driven evidence from Chinese university students—an important group experiencing rapid dietary transitions—supporting the value of a dietary-pattern framework for characterizing diet–sleep relationships beyond single foods or nutrients. Notably, PCA1 and PCA2 showed null findings, suggesting that not all empirically derived patterns are equally informative for sleep quality. One explanation is that PCA1/PCA2 may capture more heterogeneous dietary behaviors, in which potentially beneficial and less beneficial components co-occur, attenuating net associations at the pattern level.

To evaluate the stability of the PCA3–sleep association and explore potential heterogeneity, we conducted subgroup analyses stratified by sex, BMI category, disordered eating risk, and psychological distress. The inverse association between PCA3 adherence and poor sleep quality was generally consistent across strata. Although the association appeared stronger among participants with overweight/obesity, OR = 0.61 (95% CI: 0.43 to 0.88), formal tests for interaction did not provide strong evidence of effect modification; therefore, subgroup differences should be interpreted cautiously. One plausible explanation is that individuals with higher BMI may have a higher baseline burden of sleep disturbance (46) and may be more susceptible to inflammatory and metabolic perturbations that adversely affect sleep regulation (47). Dietary patterns emphasizing fresh fruits, vegetables, and dairy could be more strongly associated with sleep in metabolically vulnerable groups through their association with inflammatory and glycaemic regulation, but this hypothesis requires and biomarker-based evaluation.

Several biologically plausible pathways may link higher PCA3 adherence to more favorable sleep profiles. PCA3 emphasizes fruit- and vegetable-rich foods, which may contribute to higher intakes of dietary fiber and (poly)phenols and has been linked in prior work to lower oxidative stress and systemic inflammation (48, 49); inflammatory processes have also been implicated in sleep dysregulation (20). Higher-fiber dietary patterns may additionally influence sleep via microbiome–gut–brain signaling, including short-chain fatty acid production and downstream neuroendocrine effects (50). Beyond biological pathways, psychosocial mechanisms may also be relevant in university settings. Although we adjusted for DASS-21 and disordered eating risk, healthier eating patterns may co-occur with unmeasured aspects of self-regulation and health literacy (e.g., more regular sleep schedules, lower late-night screen exposure, or more structured daily routines). Thus, while the persistence of the PCA3 association after adjustment indicates that measured psychological distress and disordered eating do not fully account for the relationship, residual confounding by correlated health behaviors cannot be excluded.

This study has several strengths. It included a relatively large sample of Chinese university students, and standardized instruments were used to assess diet (FFQ), sleep quality (PSQI), psychological status (DASS-21), and disordered eating risk (EAT-26). The a posteriori dietary-pattern approach captures habitual dietary structure more realistically than single-food or single-nutrient analyses, and the PCA3 findings were supported by consistency across modeling strategies (continuous score and quintiles with a dose–response trend) and concordant associations across multiple PSQI components, strengthening internal coherence. Several limitations should be acknowledged. The cross-sectional design limits causal interpretation and reverse causation cannot be excluded. Dietary intake was assessed as frequency of broad food groups over the past year without portion size, processing, or timing information, and the dietary exposure window was not aligned with the PSQI recall period, which may have introduced measurement error. Although we adjusted for multiple covariates, residual confounding cannot be ruled out, and recruitment from two universities in one region may limit generalizability. The limited sample size of ethnic minorities may impact the generalizability of the study results. This will be further investigated in future research.

## 5 Conclusion

Among young Chinese university students, higher adherence to the PCA3, characterized by higher intakes of fresh fruits, fresh vegetables, and dairy products, was associated with lower odds of poor sleep quality. Prospective studies with more detailed dietary assessment (including portion sizes and food processing) are warranted to confirm these findings and to clarify the temporal relationship between dietary patterns and sleep outcomes. If corroborated, these results may inform dietary strategies to support sleep health among Chinese university students.

## Data Availability

The raw data supporting the conclusions of this article will be made available by the authors, without undue reservation.
