# Supplementary material for: Unveiling the relationship between dietary patterns and sleep quality: cross-sectional evidence from university students in Jinan city
Source: Front Nutr. 2026 Apr 2;13:1797372. doi: 10.3389/fnut.2026.1797372 (PMC13083010; doi:10.3389/fnut.2026.1797372)

Supplementary Information

Unveiling the Relationship Between Dietary Patterns and Sleep Quality: Cross-sectional Evidence from University Students in Jinan City

Table S1 Validity assessment for exploratory factor analysis (Kaiser-Meyer-Olkin and Bartlett’s Test).

Table S2 Principal dietary pattern factor loading matrix derived from principal component analysis and maximum variance rotation.

Table S3 Association between food consumption frequency (measured in days per week) and Sleep Quality (PSQI).

Table S4 Sensitivity Analysis of the association between PCA3 adherence and sleep quality.

Figure S1 Scree plot of dietary patterns derived from principal component analysis.

**Table S1** Validity Assessment for Exploratory Factor Analysis (Kaiser-Meyer-Olkin and Bartlett’s Test)

| Measure | Value |
| --- | --- |
| KMO Measure of Sampling Adequacy | 0.819 |
| Bartlett’s Test of Sphericity (χ^2^) | 4717.030(df=66,*P*＜0.001) |

**Table S2** Principal Dietary Pattern Factor Loading Matrix Derived from Principal Component Analysis and Maximum Variance Rotation.

|  | PCA1 | PCA2 | PCA3 |
| --- | --- | --- | --- |
| Rice | 0.059 | 0.686 | 0.033 |
| Wheat products | 0.320 | 0.425 | 0.111 |
| Other staple foods | 0.527 | 0.273 | 0.200 |
| Meat | 0.070 | 0.777 | 0.226 |
| Poultry | 0.169 | 0.768 | 0.152 |
| Fish/seafood | 0.689 | 0.221 | 0.186 |
| Eggs | 0.190 | 0.277 | 0.572 |
| Fresh vegetables | -0.017 | 0.380 | 0.719 |
| Fresh fruits | 0.169 | -0.076 | 0.789 |
| Dairy products | 0.339 | -0.059 | 0.694 |
| Preserved vegetables | 0.779 | 0.089 | 0.081 |
| Other dairy products | 0.798 | -0.019 | 0.164 |

**Table S3** Association Between Food Frequency Questionnaire (FFQ) and Pittsburgh Sleep Quality Index (PSQI).

|  | The group with good sleep quality  mean (SD)，N=1093 | The group with poor sleep quality  mean (SD)，N=404 | Crude Model OR (95%CI) | Model1 OR (95%CI) | Model2 OR (95%CI) |
| --- | --- | --- | --- | --- | --- |
| Rice | 2.62(0.95) | 2.64(0.97) | 1.01(0.90,1.14) | 1.03(0.91,1.17) | 1.13(0.97,1.31) |
| Wheat products | 2.44(0.92) | 2.43(0.91) | 0.99(0.88,1.12) | 0.99(0.87,1.12) | 1.04(0.89,1.21) |
| Other staple foods | 1.66(1.02) | 1.48(1.09) | 0.84(0.75,0.94) | 0.84(0.74,0.94) | 0.93(0.81,1.07) |
| Meat | 2.74(1.01) | 2.68(1.01） | 0.95(0.85,1.06) | 0.94(0.83,1.06) | 0.95(0.82,1.09) |
| Poultry | 2.50(0.99) | 2.43(0.99） | 0.93(0.83,1.04) | 0.91(0.80,1.02) | 0.94(0.81,1.08) |
| Fish/seafood | 1.34(1.02) | 1.12(0.93） | 0.80(0.71,0.90) | 0.78(0.69,0.89) | 0.91(0.79,1.06) |
| Eggs | 2.34(1.08) | 2.06(1.08） | 0.79(0.71,0.88) | 0.76(0.68,0.85) | 0.82(0.72,0.94) |
| Fresh vegetables | 2.83(1.01) | 2.69(0.99） | 0.87(0.78,0.97) | 0.84(0.75,0.95) | 0.97(0.84,1.11) |
| Fresh fruits | 2.52(1.00) | 2.18(0.98） | 0.72(0.64,0.81) | 0.69(0.62,0.78) | 0.81(0.71,0.94) |
| Dairy products | 2.26(1.05) | 2.07(1.03） | 0.84(0.75,0.94) | 0.83(0.74,0.93) | 0.91(0.80,1.04) |
| Preserved vegetables | 1.08(1.05) | 1.00(1.04） | 0.94(0.84,1.05) | 0.94(0.84,1.05) | 1.03(0.90,1.18) |
| Other dairy products | 1.04(1.12) | 0.90(1.07) | 0.89(0.80,0.99) | 0.89(0.80,0.99) | 0.96(0.85,1.10) |

Model 1 adjusted for sex, age, ethnicity, and education. (b) Model 2 additionally adjusted for smoking status, alcohol consumption, household income, household size, BMI, and EAT-26 and DASS-21 scores. Q1-Q5 represent quintiles of dietary adherence (Q5 highest).

**Table S4** Sensitivity Analysis of the Association Between PCA3 Adherence and Sleep Quality

|  | Number of participants  （N=1497） | Crude Model*β*(95%CI) | Model1 *β*(95%CI) | Model2 *β*(95%CI) |
| --- | --- | --- | --- | --- |
| PCA3 mean (SD) | 0.00(1.00) | -0.90(-1.16,-0.64) | -0.97(-1.23,-0.71) | -0.52(-0.75,-0.29) |
| PCA3 (quintiles),n(%) |  |  |  |  |
| Q1 | 300(20.04%) | 1.0 | 1.0 | 1.0 |
| Q2 | 299(19.97%) | -1.63(-2.45,-0.81) | -1.63(-2.44,-0.81) | -0.95(-1.66,-0.23) |
| Q3 | 299(19.97%) | -1.30(-2.12,-0.49) | -1.39(-2.20,-0.58) | -0.50(-1.22,0.21) |
| Q4 | 299(19.97%) | -1.87(-2.69,-1.05) | -2.04(-2.85,-1.22) | -1.22(-1.94,-0.50) |
| Q5 | 300(20.04%) | -2.88(-3.69,-2.06) | -3.02(-3.84,-2.21) | -1.70(-2.43,-0.98) |
| *P*-for-trend |  | ＜0.0001 | ＜0.0001 | ＜0.0001 |

Model 1 adjusted for sex, age, ethnicity, and education. (b) Model 2 additionally adjusted for smoking status, alcohol consumption, household income, household size, BMI, and EAT-26 and DASS-21 scores. Q1-Q5 represent quintiles of dietary adherence (Q5 highest).

**Figure S1** Scree plot of dietary patterns derived from principal component analysis


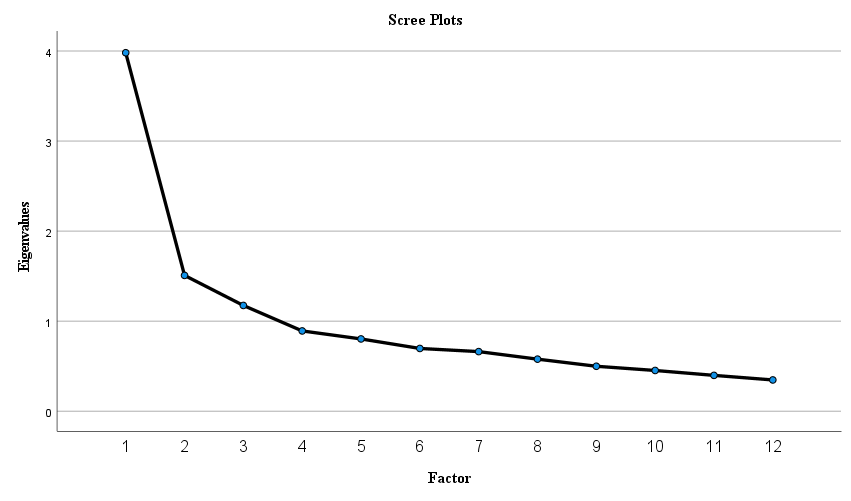

Supplement: Supplementary file 1 [file Table_1.docx]
